# A Neuro-Inspired Spike-Based PID Motor Controller for Multi-Motor Robots with Low Cost FPGAs

**DOI:** 10.3390/s120403831

**Published:** 2012-03-26

**Authors:** Angel Jimenez-Fernandez, Gabriel Jimenez-Moreno, Alejandro Linares-Barranco, Manuel J. Dominguez-Morales, Rafael Paz-Vicente, Anton Civit-Balcells

**Affiliations:** Deparment of Computer Architecture and Technology, Universidad de Sevilla, ETSI Informátca, Avd. Reina Mercedes s/n, 41012 Sevilla, Spain; E-Mails: gaji@atc.us.es (G.J.-M.); alinares@atc.us.es (A.L.-B.); mdominguez@atc.us.es (M.J.D.-M.); rpaz@act.us.es (R.P.-V.); civit@atc.us.es (A.C.-B.)

**Keywords:** neuro-controllers, neuromorphic engineering, bio-inspired systems and control, control system analysis, programmable logic devices, pulse frequency modulation, sensor-motor integration

## Abstract

In this paper we present a neuro-inspired spike-based close-loop controller written in VHDL and implemented for FPGAs. This controller has been focused on controlling a DC motor speed, but only using spikes for information representation, processing and DC motor driving. It could be applied to other motors with proper driver adaptation. This controller architecture represents one of the latest layers in a Spiking Neural Network (SNN), which implements a bridge between robotics actuators and spike-based processing layers and sensors. The presented control system fuses actuation and sensors information as spikes streams, processing these spikes in hard real-time, implementing a massively parallel information processing system, through specialized spike-based circuits. This spike-based close-loop controller has been implemented into an AER platform, designed in our labs, that allows direct control of DC motors: the AER-Robot. Experimental results evidence the viability of the implementation of spike-based controllers, and hardware synthesis denotes low hardware requirements that allow replicating this controller in a high number of parallel controllers working together to allow a real-time robot control.

## 1. Introduction

Throughout evolution, live organisms have developed efficient mechanisms in order to solve the problems they had to face in their environment. Bio-inspired systems try to understand real biological systems, extracting from them as many advantages as possible to implement them in engineering, and mimicking biology in its efficient solutions. Living beings' nervous systems in general and neurons in particular, represent the natural computing that is the inspiration of neuromorphic engineers. They work on the study and development of new computational systems inspired by the behavior of the nervous system. Recent robotic systems evidence innumerable advances in a wide variety of fields, although it is still hard for robots to interact dynamically with their environment, and in most cases, these environments must be controlled. If living beings can easily solve these questions, how can we model reality or can we model a living being's behaviors using digital devices governed by sequential or parallel algorithms? While a multiple motor controller for complex robots requires a relatively high number of computational resources (possible with currently available devices); modeling, adapting and interacting with the environment in robotics requires a computational load that cannot be achieved in the present days. One possible solution might be to progressively replace robots' controls and sensors with new neuro-inspired controls and sensory systems, trying to reproduce high level cognitive skills.

Neuromorphic systems are thought to provide a high level of parallelism, interconnectivity, and scalability, performing complex processing in real time, with a good relation between quality, speed and resource consumption. There are many researchers in neuromorphic engineering, joining together several specialist fields (biology, psychology, engineering, physics, informatics, *etc*.) in order to develop auto-reconfigurable neuro-inspired systems. Neuromorphic engineers work on the study, design and development of neuro-inspired artifacts developed with artificial devices, like VLSI chips for sensors [1–3], bio-inspired systems for processing, filtering or learning [4–8], adaptive controllers inspired in central pattern generators (CPG) [9], neuro-inspired robotics [10] and so on. There is a growing community of neuromorphic engineering researchers, as demonstrated by the success in recent years of the Telluride Neuromorphic Engineering Workshop [11] and the CapoCaccia Cognitive Neuromorphic Engineering Workshop [12].

**Figure 1.**
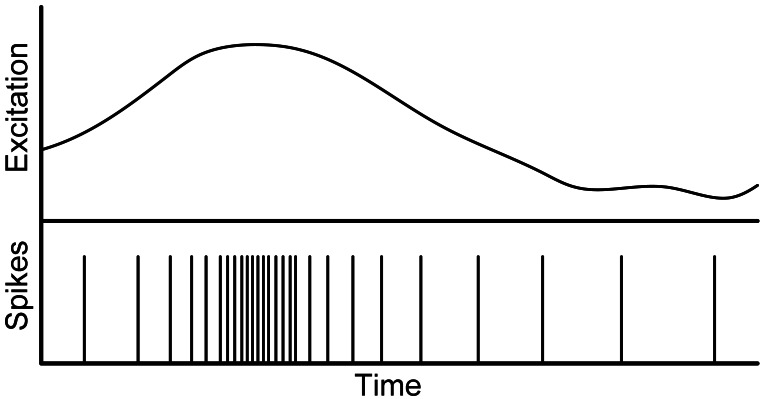
Spikes codification for a variable excitation level.

One of the neuro-inspired models, which mimics the neuron layers in the brain, is the spiking neurons model [13,14]. Spiking neurons are excited by streams of pulses (spikes), and their output is just another stream of spikes. Output spike rate is proportional to the neuron excitation, following a Pulse Frequency Modulation (PFM) scheme. For example, in vision, there are several implementations of silicon sensors that work, like in retinas, as neuro-inspired vision input layers, where an equivalent pixel is composed of a circuit whose output is a stream of spikes with a frequency proportional to a function of the illumination level. Figure 1 shows an example of a signal codified with spikes; excitation signal is presented at figure top, and the spikes that represent excitation signal at the bottom of the figure. There are several information spike-based codifications, like first-to-spike, temporal differential spike, rate coded, … In rate coded, information is codified using a PFM scheme, when excitation is low, spike rate is low and thus the time between spikes is high; however, when signal excitation increases, the inter-spikes interval time (ISI) decreases, while spike rate increases.

Spike-based information representation is a very efficient transmitting and processing information system because of several issues. Firstly, spike-based codification presents high noise immunity, because the information resides on whether or not there is a spike and in how many of them are transmitted, managing only digital levels. Secondly, this mechanism also minimizes the physical number of connections needed to communicate information between neurons to a single wire or a single virtual connection, because information is transmitted in a serial shape. In fact, solutions like AER take advantage of the relative low speed of spike streams in order to multiplex in time a set of spike streams emitters sharing a common digital bus. Finally, as the spike-based information representation could be seen as a PFM, and in this case, information cannot be periodically sampled because every spike counts, the information is continuous, not discrete. This means that there is no sample time, or global clock, that provides a constant sample rate. Consequently, this leads to processing the information spike by spike, using simple computational elements, which perform operations over spikes that do not need complex floating-point hardware or resource sharing as we will discuss later. Like biological neurons, this hardware simplicity allows the replication of computational elements, providing hardware dedicated to a specific task and the possibility of implementing a massively parallel computational model.

Current neuromorphic chips can be seen as a set of layers equivalent to those present in a mammalian nervous system: sensors, processing layers and learning. A brain receives information from several sets of sensors and it fuses this information with other cognitive and past information, it processes that information and it makes decisions about giving orders to the body for movement execution. These movements are normally received by the spinal cord as simple orders that require a set of complex movements along the muscles in the body.

In robotics, engineers typically use a CAN (Controller Area Network) for communicating with actuators and sensors, and PWM (pulse width modulation) for powering the actuators from a digital system. The use of CAN controllers implies a time cost for communicating the orders from the controller to the actuators. Furthermore, PWM also implies a delay, because the new order cannot be applied to a motor until the current period of the PWM signal passes. These delays facilitate over-dumped responses of the motor in fast responses [15]. In this work we propose the use of PFM for avoiding these delays. These digital systems are usually based on microcontrollers or embedded computers that execute low level instructions in sequence for updating the state of a motor under control. When several motors are controlled by the same microcontroller, the execution time and hardware resources are shared. These microcontrollers usually include specific hardware able to generate PWM signals. This specific hardware is based on timers, whose bit resolution (8 to 16 bits) for determining both the period of the signal and the duty cycle, lacks one of the parameters: a low resolution PWM (8-bit) could generate high frequency signals but with low precision duty cycle (256 subdivisions); and higher resolution PWM (16 bits) cannot generate high frequency signals, but it has high precision duty cycle (65,536 subdivisions). In contrast, if PFM is used, the frequency of the systems is only limited by the frequency of the input signal, and the duty cycle would be limited by the motor driver (optoacoplator and natch bridges) which implies a low pass filter. As we show in the Results section of this paper, PFM has more advantages than PWM for motor control. On the other hand, the use of microcontrollers implies resource sharing, which is not desirable for multi-motor controllers. We propose to use FPGA prototyping for implementing PFM based controllers for multiple motors in parallel as a previous step to custom chip fabrication. In this case, the power consumption will be also compared to the digital part. Nevertheless, the use of PFM instead of PWM considerably improves the power consumption when driving the motors because PFM, in average, will produce a lower commutation rate on the power stages. This is because PWM has a constant commutation rate while with PFM the commutation depends on the input of the system, thus it can be adjusted for low power.

If we think of a robot fully governed by spikes, a mechanism is needed to interface spikes information from processing layers to physical actuation elements. In this paper we present a set of mechanisms for driving, measuring and finally controlling a DC motor only with the use of spikes. In particular, this paper focuses on the design of a spike-based Proportional-Integrative-Derivative (PID) controller. This controller represents the last layer in a spiking neural network (SNN) that powers motors using spikes as in a mammal nervous system, and it also translates robot sensors information into spikes, in order to feedback this last layer and/or any other layer of the SNN.

Our spike-based PID controller approach is very simple compared to the predictive controller that seems to be implemented in the hypothalamus [16]. However it represents a significant advance in the development of spike-based robotic controllers, and another step in the direction to the design of true predictive controllers that should take the advantages of the brain inspiration: spikes coding and spikes processing of information.

In Section 2 we present the global idea behind the spike-based PID controller, and the way we develop spike-based controllers with building blocks. Section 3 presents the experimental setup that we have used to test the spike-based PID controller in a real scenario with a commercial DC motor. In Section 4 experimental results are presented and discussed. Then, in Section 5 we present the hardware requirements and operating frequencies after synthesizing diverse spike-based PID controllers. Finally, conclusions are presented in Section 6.

## 2. Spike-Based PID Controller Design Overview

This section presents details about the rotation speed of a DC motor, but it can be applied to other kind of motors through the proper adaptation. A brushed DC motor is relatively simple to power since only one signal with a voltage in between the range of the motor is enough to make it run. But there are different motors that need more logic to be managed properly, like for example a brushless motor or a step motor. Normally a brushless motor needs three different non-continuous signals to make the motor run. These signals have to be periodic and with different phases, which reminds one of spikes. Therefore, a spike-based motor controller could be applied to any motor using the proper adaptation that does not require delays.

In control theory, the general idea of a closed-loop controller is to compare the real system state with a desired system state, getting the system error. This system error value is processed, and then applied to the system under control. A classic type of closed-loop controllers is the Proportional-Integrate-Derivate (PID). This controller calculates three components: (1) one is proportional to the system error; (2) another one is proportional to the temporal integration error; and (3) the last one is proportional to the temporal derivative error. The addition of these three components is the value of the signal used as the input of the system under control, called actuation variable. PID controllers can be implemented as analog circuits, based on continuous system modeling (using e.g., Laplace transform), or as digital circuits, modeling a discrete controller (using e.g., the discrete Z-transform). Discrete closed-loop PID controllers are currently implemented in a wide diversity of digital devices, like Digital Signal Processors (DSP), microcontrollers, or embedded processors inside FPGAs. Usually these systems execute PID-controllers as sequential algorithms, which generate quantized output samples with fixed sample time. All these different architectures always need complex general purpose hardware to perform Multiplicative-and-Accumulative (MAC) operations, wide buses to allow the communication between operational units and processor registers, and they also demand shared resources, such as memory. Consequently, additional mechanisms, at different levels, should be implemented in order to multiplex in time hardware units and share resources for every discrete controller when many of them must be synthesized or implemented in parallel. This leads to a sequential and blocking resources access. For these reasons, it is difficult to implement a high number of digital real-time controllers running fully in parallel inside a single device.

A spike-based PID-controller is very different to the discrete ones mentioned here. As we will see later, it is closer to a continuous analog controller, but using fully digital circuits. Analog PID controller implementation performs control operations over analog signals, and the hardware elements which perform these operations are usually operational amplifiers (OA), and passive components such as resistors or capacitors. These components perform basic operations over analog signals. We propose to develop similar controller architectures but using only spikes (both internally and externally). In order to achieve this it is necessary to design hardware components that perform the same basic operations used in analog circuits, but over spike streams.

**Figure 2.**
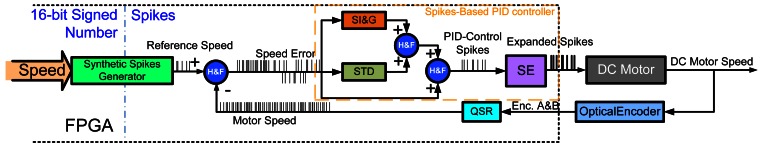
Spikes-based PID controller internal building blocks.

Figure 2 shows our spike-based PID controller, with its internal blocks. There are only spikes flowing between these blocks, being processed while they flow, until they are applied to a motor (DC motor in our tests). As neurons are small specialized computational units that perform specific operations, the spike-based PID-controller has been constructed using small hardware units designed as building blocks. Since each controller is using basic digital components (counters, registers and comparators), it is feasible to implement many PID controllers in the same FPGA (or chip) working in parallel without resource sharing. Each spike processing building-block has to provide a new stream of spikes that modifies the spike frequency according to a specific mathematical primitive operation, which can be addition/subtraction, integration and derivation for our case. Other elements are mandatory, as the conversion of motor/robot sensor information into a spike coded stream for the close-loop; or, in the case of brushless motors or steps motors spikes produced, these cannot be applied directly to the motors, and special bridges are needed for changing accordingly the phases to be applied to the motors. Throughout this paper all the building blocks included in the spike-based PID controller will be explained in depth. These elements have been written in VHDL, which can be synthesized as digital circuits for FPGAs, and they can be used as building blocks for larger systems. Although in this paper we present a particular spike-based PID controller, designed building blocks allow to reuse and combine them in different ways, giving us the opportunity to build a diverse kind of systems for Spikes Signal Processing (SSP), like for example spike-based filters [17]. Spike processing building blocks are composed of dedicated hardware that works independently from each other, and thus, when synthesizing several of them on the same FPGA, they can behave as a parallel processing system.

From a system level point of view, the idea behind the spike-based PID controller is to use a spike stream in order to achieve a fixed speed of rotation of a motor with a closed-loop controller. In this work, a reference spike-based signal is generated using a synthetic spikes generator (see Figure 2, left). From these reference spikes, in order to design closed-loop control systems, those spikes that codify the real motor speed should be subtracted (left H&F on Figure 2). The subtraction of the real speed from the reference, both of them codified into spikes, will provide a new spike stream that codifies the system error, in the same way as traditional closed-loop systems. Those error spikes will be processed by several building blocks while they are flowing through the controller until they are applied to the motor. While spikes are flowing through the controller, integrative and derivative operations are performed over the error spikes. And this implies the computation implemented by the PID controller. The provided output is equivalent to a traditional continuous PID controller, but using spikes and it is implemented on digital devices such as FPGAs instead of analog devices.

### 2.1. Spikes Expansor (SE)

The first question we have to face in order to develop spike-based controllers is to find out how to implement an interface between the controller output spikes and the controlled system input. The adequate mechanism for this used to be application dependent. In our case, we want to focus our attention on Spike-PID controllers applied to robotics, as in this paper the controlled system will be a DC motor as part of a robot. Therefore, the mechanism that we will propose in this paper is focused on driving DC motors with spikes. Nevertheless, the same controller spike-based output can be applied to any other motor (brushless, step, …) simply by modifying or inserting adequate bridges.

In robotic applications it is very common to drive DC motors from digital circuits using Pulse Width Modulation (PWM). PWM applies to a DC motor a periodically sampled signal, whose sample period (T_PWM_), is constant, but its high time (T_h_), or duty-cycle, is variable. The information, or power value applied to the DC motor, resides in the PWM signal duty-cycle. Thanks to the fact that a DC motor works like a low-pass filter, it rejects the PWM signal high frequency components and it only retains the DC and low frequency components. Thus, from the motor perspective, DC power (V_DCmotor_) is proportional to the PWM signal duty-cycle, as indicated by Equation (1), where V_PS_ is the DC voltage that is provided by the power supply stage:

(1) $$V_{\text{DCmotor}}\approx \frac{T_{h}}{T_{\text{PWM}}}V_{\text{PS}}$$

In the context of spikes, the information, or in this case DC motor power, is codified following a Pulse Frequency Modulation (PFM) approach where information resides in the spikes' frequency. In a PFM scheme the modulated signal high time (T_h_) is fixed, and the frequency, or Inter-Spikes-Interval (T_ISI_), is variable, as shown in Equation (2). Therefore, PFM and PWM can be seen as opposite schemes. Equation (2) is equivalent to Equation (1), but adapted to PFM modulation. Equation (2) evidences that PFM modulation is equivalent to PWM, but DC power is controlled with the spike rate. Thereby, we propose to drive DC motors directly with spikes. This is an alternative to PWM systems, but without an intrinsic sampling period:

(2) $$V_{\text{DCmotor}}\approx \frac{T_{h}}{T_{\text{ISI}}}V_{\text{PS}}=T_{h}*{\text{Spikes}}_{\text{rate}}*V_{\text{PS}}$$

**Figure 3.**
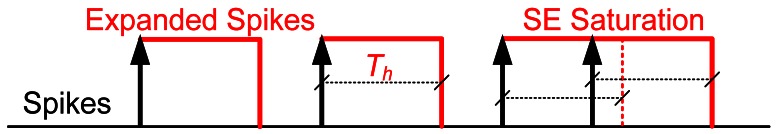
Expanding spikes in order to drive a DC motor.

If we look carefully at Equation (2), we can realize that if spikes are very short in duration (e.g., 20 ns in our system), they do not represent much energy and, thus, it will be filtered by the power stage (optoacoplators and/or natch bridges). In the best case, spikes will be filtered by the DC motor, demanding a very high spike rate in order to provide the DC motor with energy for normal operation. The solution adopted in this work for providing more energy per spike is to increase their width by a fixed time (e.g., T_h_ = 1 μs). As we can find in [18], T_h_ affects the DC motor static gain, or in other words, setting up the high time associated to each spike, we can control how much power is applied to the DC motor by one spike. It is clear that the higher T_h_ is, the greater the static gain will be. Figure 3 shows how incoming spikes (black) are expanded in time (red). A non-linear effect of the spike expansion is the saturation as shown in Figure 3, right. For example, if we increase a spike 1 μs, and before this time ends, a new spike arrives, this second spike will be increased again fixing the output to high level for 1 more μs.

**Figure 4.**
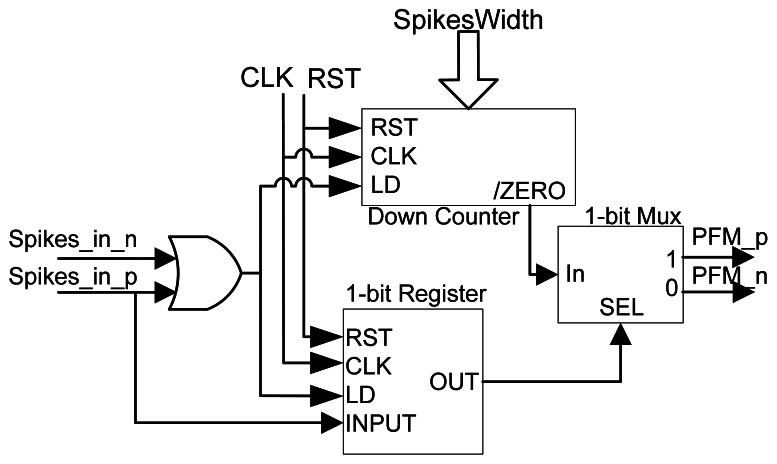
Spikes Expansor internal components.

In order to perform this operation, we have designed a configurable element that allows expanding spikes to a fixed, but parameterized time that we have called *SpikeExpansor* (SE purple box on the right side of Figure 2). Figure 4 shows the SE internal circuit, based on a digital down counter with auto-stop and an active-low ZERO output signal. The idea is to set the down counter with a fixed value, *SpikesWidth*, and count down as many clock cycles as indicated by this value. While the down counter is being decremented, ZERO, which represents the expanded spike, will be set to ‘1’. In order to manage signed spikes that transmit positive or negative voltage to the DC motor, some additional hardware should be added. This hardware consists of a 1-bit register and a 2-channel multiplexor. The aim of these elements is to record the last spike sign received, and transmit the expanded output spike to the right signal in order to provide positive (PFM_p) or negative (PFM_n) spikes. The final power stage, responsible for powering the DC motor, should be able to provide positive and negative power to the DC motors, like for example a full H-bridge.

Taking into account SE features we can set up a desired T_h_ using the *SpikesWidth* signal according to Equation (3), where T_h_ is proportional to *SpikesWidth* and the FPGA clock period (T_CLK_):

(3) $$T_{h}=T_{\text{CLK}}*\text{SpikesWidth}$$

Using Equations (2) and (3) we are able to calculate the equivalent SE static gain for each spike, considering that the equivalent SE static gain can be set with *SpikesWidth* signal value:

(4) $$k_{\text{Static}}=T_{\text{CLK}}*\text{SpikesWidth}*V_{\text{PS}}$$

### 2.2. Quadrature Encoder to Spike Rate (QSR)

**Figure 5.**
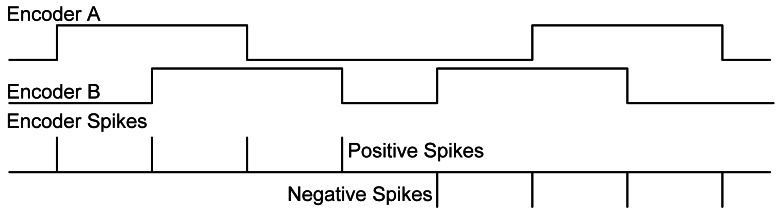
Spikes coming from a quadrature encoder.

Once we know one possible mechanism to drive a motor with spikes, the next question is how to measure a motor rotation speed with rate coded spikes. A very common sensor used to measure motor speed accurately, is the quadrature encoder. Typical quadrature encoder produces two signals, called A and B, with a 90° phase difference (see Figure 5, top). In both signals the motor sensed speed is coded by a 50% duty-cycle square signal. The instantaneous frequency of these signals represents the motor speed. A and B signals follow a PFM scheme. In other words, the motor speed is codified, in A and B, by pulses frequency. To perform the translation from quadrature optical encoder speed information (contained in the frequency of A and B signals) to a spike stream, we have designed a building block, the Quadrature Encoder to Spike Rate (QSR) converter [19], in Figure 2 clear blue on the feed-back from the motor.

In Figure 5 we show the expected behavior of QSR. The top of the figure top shows A and B signals coming from a quadrature encoder. Previously we have commented how this device usually codifies the speed into frequency; however the direction of the motor rotation, forward or backward, is codified in the relative phase between A and B signals. For example, in Figure 5, the DC motor starts rotating forward, because A goes high before B. However, later DC motor rotates backward, and then B goes high before A, indicating that it is rotating at the opposite direction.

The translation from quadrature encoder speed information into spikes consists simply of firing a positive or negative spike for every edge on the A or B signals. This sign depends on the rotation direction. This functionality is provided by QSR using a finite state machine.

Another possible solution for the feed-back sensing on spikes, but without the use of any specialized sensor, is to use the motor in generator mode. This mode could be used in order to get the speed state of the motor instead of using quadrature encoders. Nevertheless, quadrature encoders are more precise because they are measuring the speed continuously but the generator mode of a motor implies to measure the speed only in those phases or times when no power is provided to the motor, which is easier for PWM because it is possible to use a time slice just before the next duty cycle in order to measure those currents produced in the brush during the rotation of the motor. But with PFM these time slices are not predictable.

### 2.3. Reverse Bit-Wise Synthetic Spikes Generator (RB-SSG)

Using the previously proposed spike processing building blocks (SE and QSR) we are able to implement an interface between a DC motor and a spike-based controller. The next question to solve is how to generate spikes in order to stimulate the inputs of our spike-based controller. To achieve this we propose a digital Synthetic Spikes Generator (SSG) that is able to convert a digital word (SSG input) into a fixed spike frequency rate (SSG output) (Figure 2, green rectangle on the left). These spikes will codify the target speed of the motor. However, this element will not be used only as the generator of stimulus spikes, but it will also be part of other spike-based building blocks that will be presented in next subsections. Although there are several ways to design a digital SSG [20], we use a SSG based on the reverse bitwise method for synthetic AER event generation, which can be found in [21]. This architecture has been selected mainly because of its hardware simplicity: basically it only needs digital counters and comparators and it has a good hardware resource consumption and temporal spike distribution ratio.

In general, an RB-SSG should generate a synthetic spike stream, whose frequency should be proportional to a constant (k_BWSpikesGen_) and an input value (x), according to Equation (5):

(5) $$\text{RB}-\text{SSG}{\left(x\right)}_{\text{SpikesRate}}=k_{\text{BWSpikesGen}}*x$$

**Figure 6.**
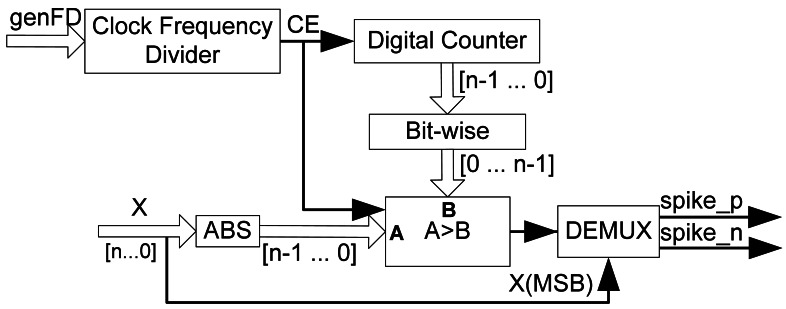
Reverse Bitwise Synthetic Spikes Generator internal blocks.

Figure 6 shows the circuit of the RB-SSG. It uses a continuous digital counter (top figure), whose output is reversed bitwise and compared with the input absolute value (ABS(x)). When the input absolute value is greater than the reversed counter value, a new spike is fired (bottom figure). RB-SSG ensures a homogeneous spike distribution along time, due to the bitwise reversal output of the counter. Since the speed reference could be signed, it is necessary to generate positive and negative spikes. Therefore, we use a demultiplexor (DEMUX) to select the right output (positive or negative) for the spikes generated. DEMUX selection signal is the X input sign, or X(MSB). Finally, a clock frequency divider (left top figure) is included to adjust accurately the RB-SSG gain. The clock frequency divider will activate a clock enable (CE) signal used to divide the spike generator clock frequency, according to a frequency divider signal (genFD). Consequently the RB-SSG gain (k_BWSpikesGen_) can be calculated as in Equation (6):

(6) $$k_{\text{BWSpikesGen}}=\frac{F_{\text{CLK}}}{2^{N-1}(\text{genFD}+1)}$$

where F_clk_ represents the system clock frequency, N is the RB-SSG bit length, and genFD is the clock frequency divider value. These parameters can be modified in order to set up RB-SSG gain according to the design requirements.

### 2.4. Spikes Hold & Fire (SH&F)

At this point we have presented a set of spike processing building blocks which are able to stimulate a spike-based controller (RB-SSG), to drive a DC motor (SE), and measure rotation speed (QSR) for the controller feed-back. Next, we will introduce three spike-based building blocks that will perform the real primitive arithmetic operations over spike coded signals. These blocks use spike streams as their inputs and outputs. The first operation that we are going to perform is the subtraction between two spike streams. For this operation we have implemented a spike processing building block called Spikes Hold & Fire (SH&F), which allows to “close the controller loop” (see Figure 2, left H&F dark blue box). This block will subtract the real motor speed spikes, coming from the QSR, from the reference input spike stream (generated by RB-SSG) providing the error spikes [18]. This element is also used to add two spike streams, as we can see in Figure 2, by only inverting the sign of the spikes from negative input of the SH&F. However, this explanation is focused on spike subtracting.

Subtracting a spike input signal (f_Y_) to another (f_U_), means to provides a new spike signal which has a spike rate (f_SH&F_) that is the difference between both inputs spike rates (see Equation (7)):

(7) $$f_{\text{SH}\&F}=f_{U}-f_{Y}$$

**Figure 7.**
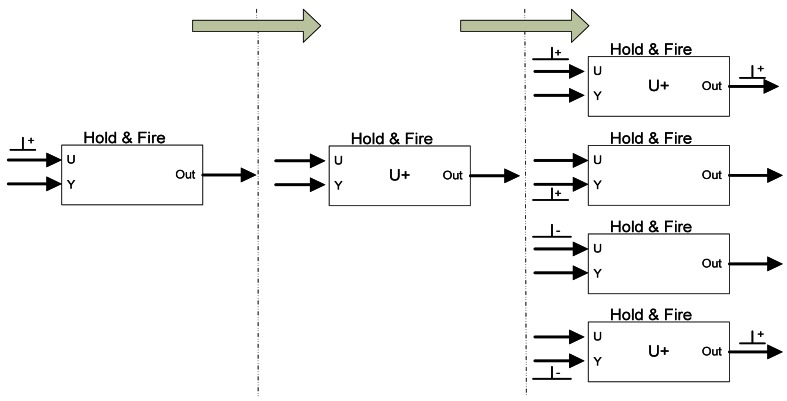
Spikes Hold&Fire evolution form a positive U spike.

The function of the SH&F is to hold the incoming spikes for a fixed period of time (in this work we have used a fixed hold time of 10 μs, but it can be modified before the synthesis. SH&F has been used in other applications with hold time from 1 μs to infinite) while it is monitoring the input spikes to decide if output spikes must be produced or not. Figure 7 shows an example of SH&F evolution from a positive input spike. SH&F has two inputs: U (positive input) and Y (negative input, commonly used as feedback). Let us suppose that a positive, U+, spike is received on U input (figure left). U+ state is held internally (center figure). SH&F will do nothing in case no more spikes are received. When a new spike arrives, it behaves according to spike input port and sign (right figure). On right top, if SH&F receives a positive spike (U+), the previously held spike is fired as a positive spike (SH&F+), and a new one is held internally (U+ state). If a negative input spike is received in the port U (U−), or a positive spike in the port Y (Y+), the previously held spike is cancelled and no spike output is produced. Finally, if a negative spike (Y−) is received in Y (bottom figure), the previously held spike is fired and the last one received is held with positive sign (U+ state). Similarly the SH&F behavior can be extended to any kind of input spikes (U−, Y+ and Y- states) using the same logic: hold, cancel, and fire spikes according to the input spike ports and sign. This block has been successfully used previously to design spike-based processing systems (SSP) like several kinds of spike-based filters [17,22,23].

**Figure 8.**
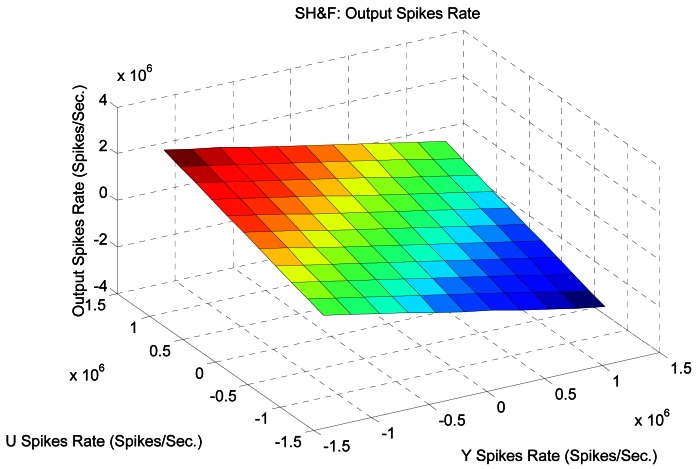
Simulated spikes SH&F output spikes rate for Y and U from −1.5 to 1.5 Mspikes/s.

In order to characterize this element we have performed a simple test where both input frequencies vary from −1.5 Mspikes/s to 1.5 Mspikes/s. Figure 8 shows the output of the SH&F for these input PFM variation both in Y and U. It can be seen graphically how the output frequency corresponds to the subtraction operation.

**Figure 9.**
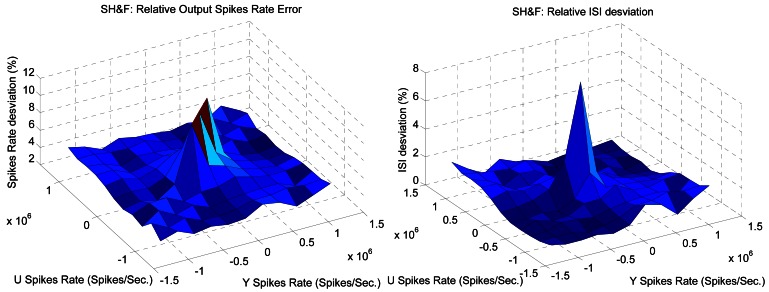
Spikes Hold&Fire standard deviation from ideal output.

Figure 9 shows how far the SH&F output frequency from the ideal subtraction is measured as the standard deviation from the ideal frequency on the left, and as the standard deviation from the ideal ISI on the right. The values have been normalized and expressed in %. In general there are low errors except when the frequencies of both inputs are low. In this case, hold time produces changes (less than 10% in the worst case) in the spike output distribution.

### 2.5. Spikes Integrate & Generate (SI&G)

**Figure 10.**
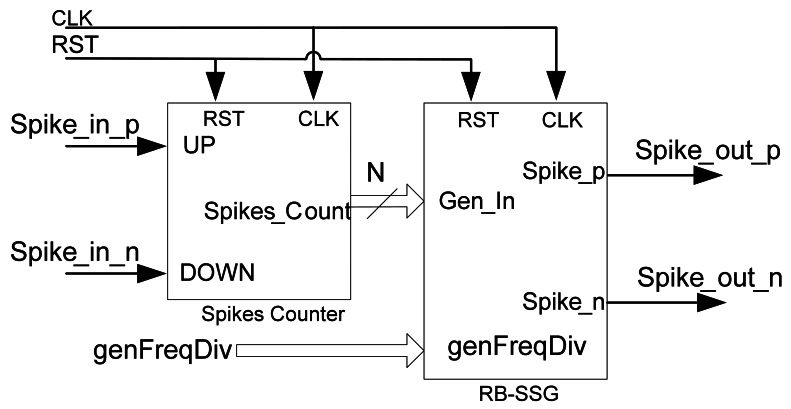
Spikes Integrate & Generate block diagram.

The next spike processing building block is an element that will perform the spike integration. This component is used in the spike-based PID controller as an integral component, and we call it Spikes Integrate & Generate (SI&G) [17] (orange in Figure 2). SI&G is composed of a spike counter and an RB-SSG, as shown in Figure 10. The spike counter is a digital counter whose value increases when a positive spike is received, and decreases when a negative spike is received. The counter output is exactly the spikes integration value, thus, in order to convert integrated count again to spikes, the spikes counter output will be connected to the RB-SSG input, and this will generate again a new stream of spikes. These spikes will have a frequency proportional to the spike count, *i.e*., the spikes integration.

Considering this architecture, the SI&G output spike frequency can be calculated as expressed in Equation (8). SI&G gain, k_SI&G_, is set using the RB-SSG parameters included. In fact it is equivalent to k_BWSpikesGen_, and can be tuned with the same parameters we previously presented in Equation (6) to set up the RB-SSG gain: N, F_CLK_ and genFD:

(8) $$f_{\text{SI}\&G}=k_{\text{SI}\&G}*\int_{0}^{t}f_{\text{inputSpikes}}\text{dt}=\frac{F_{\text{CLK}}}{2^{N-1}(\text{genFD}+1)}\int_{0}^{t}f_{\text{inputSpikes}}\text{dt}$$

Similarly to what happens in continuous systems, we can calculate the equivalent SI&G transfer function in the “spike-domain” using the Laplace transformation [24]. The SI&G transfer function is presented in Equation (9). It is equivalent to an ideal integrator with a gain of k_SI&G_, but in the context of spikes. This gain depends directly on F_CLK_ and inversely on genFD and 2^N−1^:

(9) $$\text{SI}\&G(s)=\frac{F_{\text{SI}\&G}(s)}{F_{\text{inputSpikes}}(s)}=\frac{k_{\text{SI}\&G}}{\text{s}}=\frac{F_{\text{CLK}}}{2^{N-1}(\text{genFD}+1)*\text{s}}$$

**Figure 11.**
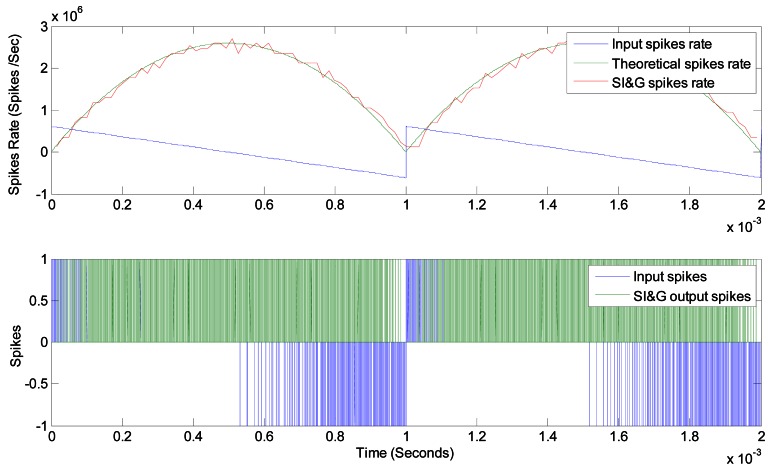
Spikes Integrate & Generate response.

Figure 11 presents an example that justifies the correct response of this block. Both input spike rate (blue) and output spike rate (green) are represented at the bottom. The same can be seen at the top of the figure, although represented in spikes rate values. Input represents a saw tooth with positive and negative values (blue). Ideal integral is presented in green and simulated output is presented in red. The correspondence can be seen graphically.

### 2.6. Spike Temporal Derivate (STD)

**Figure 12.**
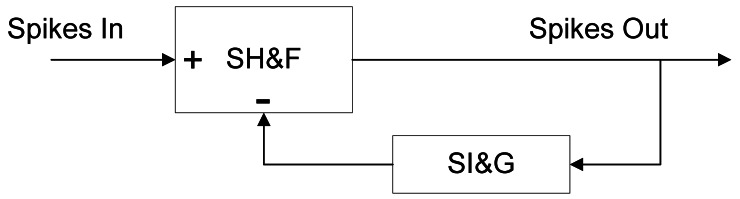
Spikes Temporal derivative architecture.

As SI&G implements the integral component of the spike-based PID controller, a spike processing building block that implements the temporal derivative of a spike rate coded signal is also necessary. For this purpose we have designed a building block that allows spike stream derivation, the Spikes Temporal Derivate (STD) building block (dark green in Figure 2). To implement STD we have used an SH&F and a SI&G as shown in Figure 12. The idea is to feedback an SH&F with SI&G, being SH&F the difference between input spikes, and the integration of derivate spikes. Spike signals that interconnect the blocks presented in Figure 12 contain both positive and negative spikes.

Applying basic systems theory, considering the topology of STD shown in Figure 12, we can calculate the STD continuous equivalent transfer function (Equation (10)) using the equivalent SI&G transfer function presented previously in Equation (9)

(10) $$\text{STD}(s)=\frac{1}{1+\text{SI}\&G(s)}=\frac{1}{1+\frac{k_{\text{SI}\&G}}{S}}=\frac{s}{s+\frac{F_{\text{CLK}}}{2^{N-1}(\text{genFD}+1)}}$$

Equation (10) indicates that STD behaves like a high-pass filter, with a zero in the origin, like an ideal derivative, and a real pole placed at k_SI&G_ (rad/s). Therefore, STD is actually not an ideal derivative (as it usually happens with equivalent analog ones) so, its behavior will be equivalent to input derivative, but with an intrinsic dynamic component. Without considering this common non-ideality, the equivalent STD gain, k_STD_, presented in Equation (10) can be set as shown in Equation (11):

(11) $$k_{\text{STD}}=\frac{2^{N-1}(\text{genFD}+1)}{F_{\text{CLK}}}$$

One important question that should be taken into account is how k_STD_ affects the STD non-ideal pole cut-off frequency. Equations (10) and (11) indicate that both STD features are inversely proportional to each other, so the greater the k_STD_, the lower the cut-off frequency, and *vice versa*.

**Figure 13.**
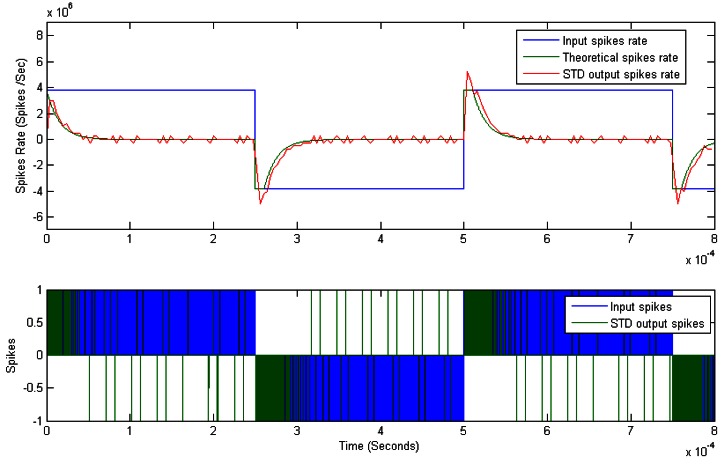
Spikes Temporal Derivative response.

Figure 13 presents an example that justifies the correct response of this block. Both input spike rate (blue) and output spike rate (green) are represented at the bottom. The same can be seen at the top of the figure, although represented in spikes rate values. Input represents a periodic square signal with positive and negative values (blue). Ideal integral is presented in green and simulated output is presented in red. The correspondence can be seen graphically.

### 2.7. Equivalent Spike-Based PID Controller

Spike-based PID controller topology was presented in Figure 2 inside an orange box. The idea is to propagate, integrate and derivate error spikes, using two SH&F for spike addition, and with SI&G and STD. In the final step, these spikes will be expanded and applied to the DC motor. We can calculate the spike-based PID controller equivalent transfer function in Equation (12) adding the transfer function of SI&G, in Equation (9), STD in Equation (10), and multiplying by the effect of spike temporal expansion, in Equation (4):

(12) $$\text{SPID}(s)=(1+\text{SI}\&G(s)+\text{STD}(s))*k_{\text{SE}}$$

The proportional component will be represented by the propagated error spikes, which will be expanded in time, with a value of k_SE_. The integral component will be composed of the integration of error expanded spikes, so integral gain will be k_SIG_ × k_SE_. Finally, the derivate component will be in the STD output spikes, and it will also be expanded, so, its gain will be k_STD_ × k_SE_. It is important to highlight that the STD cut-off frequency does not decrease with the spike temporal width. Spike-based PID controller behavior can be set up using the previously introduced spike processing building blocks parameters. These parameters are as simple as the expanded spikes high time, and the number of bits of the digital counters of diverse RB-SSG included in the blocks.

## 3. Experimental Platform: The AER-ROBOT

**Figure 14.**
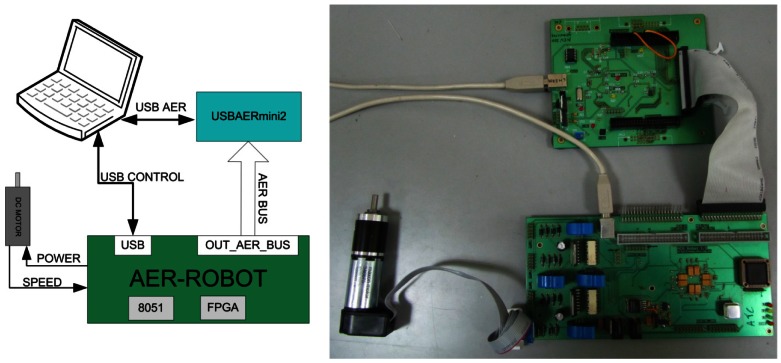
Experimental scenario: (**left**) block diagram; (**right**) photography.

After presenting how to design and tune the spike-based PID controllers, in this section we show the experimental platform used to test the spike-based PID controllers. Figure 14 left shows the block diagram of the designed experimental platform, and a real photograph is shown on the right. In order to bring the spike-based PID controllers to reality we need to meet several requirements. First of all, (a) we need a programmable logic device capable of implementing the spike-based PID controller and driving DC motors. It is also very interesting to (b) manage the spike-based PID controller parameters for experimental purposes. Finally, the (c) internal controller spike activity should be monitored, in order to analyze the spike-based controller responses off-line. With these (a), (b) and (c) purposes we have combined different neuro-inspired hardware platforms based on the use of Address-Event-Representation (AER) [25]. The combined platforms are the AER-Robot [25] (see Figure 14 right bottom) and USBAERMini2 [26] (see Figure 14 right top). Both elements were designed to work together performing different tasks. We have downloaded a configurable spike-based PID controller to the FPGA included in the AER-Robot. Since the AER-Robot architecture includes an 8051 microcontroller with USB capabilities, we managed the PID parameters from a PC through an USB port. We codified the controller reference spikes input using the USB port of the AER-Robot and an internal RB-SSG. Furthermore, the internal controller spikes can be codified as AER events and they can be monitored, for debugging purposes, from a PC through the USBAERMini2 (on monitor mode) CPLD and USB 2.0 high speed microcontroller.

The AER-Robot board will implement and manage the spike-based PID controller. This board will also drive the DC motors (up to four), and it will also transmit the controller spikes as AER events. The AER-Robot platform is a hardware/software co-design platform, which includes an FPGA and a USB 2.0 full-speed 8051 microcontroller (see Figure 14). The AER-Robot platform is a general purpose interface between neuromorphic AER systems and robotic actuators like DC motors, and it was originally designed to control an anthropomorphic robotic hand in a neuro-inspired way [25]. However, thanks to its versatility, the AER-Robot can be configured and adapted to work in diverse scenarios. Each AER-Robot is able to control up to four DC motors (and measure their encoder signals), and it also includes four AER ports for system scalability. The FPGA is connected to AER buses, to H-bridges that drive the DC motors, to the encoder channels, and to the microcontroller. The microcontroller has a USB 2.0 port and a powerful ADC that can be used for measuring other robotic sensors, like hall-effect current sensor included in the PCB. The microcontroller is connected to the FPGA through an SPI port.

In this work, while the FPGA implements the spike-based PID controllers for up to four DC motors, the microcontroller provides a USB connection to a PC for parameter managing, and it sends analog (and digital) sensor values to the FPGA. When this platform is included in a neuro-inspired multi-layer hierarchical system, it will represent an input/output layer, which will control DC motors with the AER input information. It can also provide controller state information on the AER outputs. The main features of this platform are:

- Spartan 3 FPGA (XC3S400) clocked at 50 MHz.
- 24/48-MHz 8051 microcontroller from Silicon Laboratories (C8051F320/342).
- Four AER parallel ports for full scalability.
- Four H-bridges to drive DC motors with a hall-effect current sensor and two encoder channels for every motor.
- USB 2.0 Full-Speed connectivity.
- 12 analog inputs (200k Samples—10 bits).

The USBAERmini2 [27] is used for monitoring, visualizing and analyzing the spike activity (in AER). This device allows monitoring and sequencing AER streams with a minimum time resolution of 0.2 μs. It can be inserted in between two AER devices (in pass-through mode) or it can be used in terminal mode as the last element of an AER chain, which is the case of this work. The USBAERmini2 achieves a peak monitor rate of 5 × 10^6^ Events/s and a sustained rate of 4,5 × 10^6^ Events/s, which is limited by the host computer (PC or laptop). The host software, jAER [28], provides the interface between the USBAERmini2 and MATLAB. jAER is an open-source java software for interfacing a computer to AER devices. jAER provides visualization and processing tools for AER data. The jAER classes are accessible from MATLAB, providing an easy way to stimulate, capture and analyze AER information. USBAERmini2 was designed in the frame of the Convolution Address-Event-Representation (AER) Vision Architecture for Real-Time (CAVIAR) EU project [29].

## 4. Experimental Results

This section presents experimental results obtained when a DC motor is controlled with the previously presented spike-based PID controller. For these tests we have selected a DC motor manufactured by Maxon Motors, whose features are presented in Table 1. This motor has attached an optical quadrature encoder, which will be used to feed-back the controller. It must be clear that, in this paper we do not attempt to tune or give a method for tuning a PID controller. There is an extended classical bibliography about it [24,30], however the application of bio-inspired self-tuning techniques, as in [31], to spike-based controllers would be very interesting. Our aim is to verify that spike-based PID controllers are viable and behave similarly to equivalent continuous controllers.

**Figure 15.**
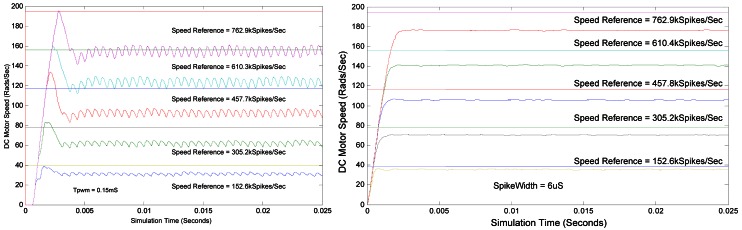
PWM (**left**) *vs*. PFM (**right**) Proportional close-loop controller results.

Figure 15 shows a comparison between a proportional controller based on PWM (left) and based on PFM (right). PWM period is fixed to 150 μs. For different reference speeds, the controller is not able to reach the objective, due to the absence of I and D components, but furthermore, there is a under-damped effect on the response due to the delays introduced by the PWM period. Nevertheless, Figure 15 right is showing similar results for PFM with a spike width of 6 μs. The objective is not met neither in this case for the same reason, but the under-damped effect is not present.

**Figure 16.**
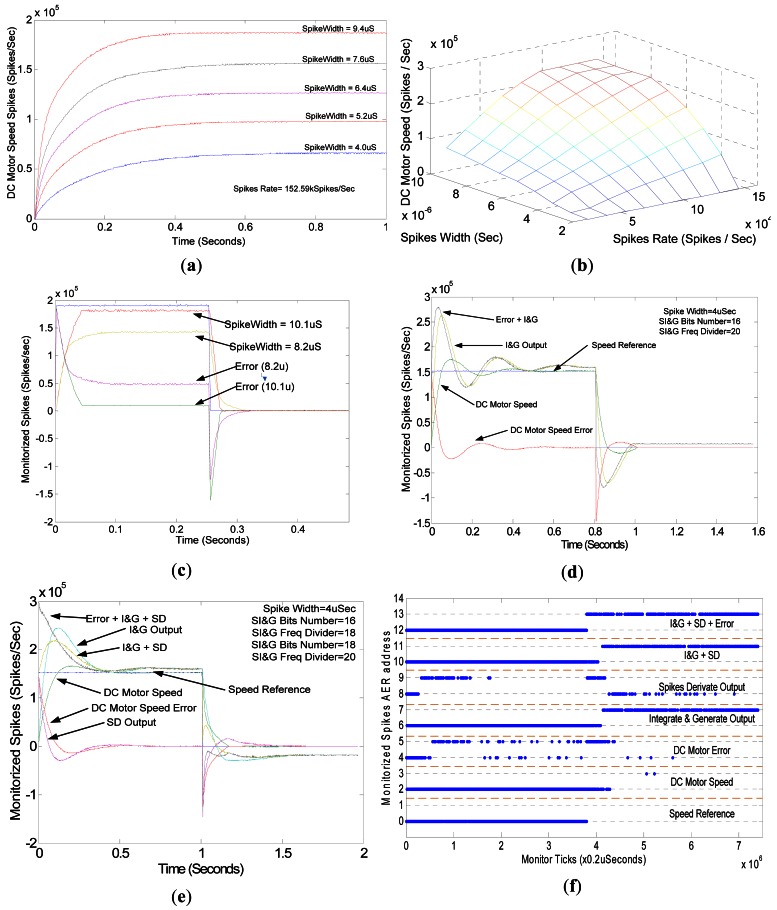
Experimental results: (**a**) Open-loop responses; (**b**) Static characteristic; (**c**) spikes-based P control; (**d**) spikes-based PI control; (**e**) spikes-based PID controller; (**f**) monitored AER events.

In Figure 16 we present the results of diverse spike-based controller settings. Figure 16(a) shows the DC motor response in open-loop, being driven directly with temporal expanded spikes. In this experiment we have stimulated the DC motor with a constant spike rate (equivalent to a continuous step) of about 152 kSpikes/s, but with different spike widths (from 4 μs to 9.4 μs). The DC motor speed has been reconstructed with the aid of the spikes fired by the QSR block, which come from the quadrature encoder [32]. The DC motor response is equivalent to a second order over-damped system that filters the spikes of high frequency harmonics and retains only the DC component. This figure shows that the DC motor reaches a speed proportional to the spikes temporal width, and consequently the static gain associated to each spike, as predicted in Equation (4).

Figure 16(b) shows the static characteristic of the experiment. In order to get the static characteristic we have made a parameter sweep, stimulating the DC motor with different spike rates and diverse temporal widths (X & Y axis), logging the DC motor steady state speed (Z axis) for every parameter pair. This plot evidences that the higher the spike rates or temporal widths are, the higher the DC motor speed is. The relation between speed and spike rate is lineal; however, regarding the spikes temporal width there is a non-linearity. Fortunately, in our spike-based controller, the spike width is a controller constant and the actuation variable is the spikes rate. The saturation is also interesting when spikes rate or spikes width are very high. In this case, SE saturates, fixing its output to ‘1’, and the maximum speed allowable by DC motor is reached.

In the next experiment, we have controlled the DC motor with a closed-loop spike-based Proportional (P) speed controller. With this purpose we have used the spike-based PID controller presented in Figure 2, but holding the SI&G and STD in rest state, and thus applying to DC motor only the spikes that represent the speed error, after being expanded. As Equation (4) evidences, with the spikes temporal width we can set the equivalent static gain related to the spikes, *i.e.*, we can implement a spike-based P controller, where the controller gain can be set with the spikes temporal width. Therefore, using Equation (4) and applying basic systems theory, the equivalent spike-based P controller gain can be calculated as follows:

(13) $$k_{P}=\frac{k_{\text{Motor}}k_{\text{Static}}}{1+k_{\text{feedBack}}k_{\text{Motor}}k_{\text{Static}}}$$

where k_Motor_ is the DC motor static gain (rads/s/V), k_feedBack_ is the relation between DC motor speed and the spike rate from the quadrature encoder (spikes/sec/rads/s) and, finally, k_Static_ can be fixed using Equation (4). This parameter only depends on the spikes temporal width. In this way, we implemented a spike-based P controller, where the P factor or controller gain k_P_, can be tuned by adjusting the spike width. Figure 16(c) shows the DC motor response, using a spike-based P controller, for two different spike temporal widths, or k_P_. In this plot we show the P controller spike frequency reconstruction, in blue we present the controller input spikes that perform an input step, colored in yellow and red are the spikes that codify the DC motor real speed, for different spike temporal widths (10.1 μs and 8.2 μs) and finally, in green and purple, the controller error in both cases. The DC motor speed presents an over-damped response, with a lower rise time when compared to the response in open-loop (from 0.4 ms to 0.05 ms), although presenting a steady state error, as it is expected from a continuous P controller. The spike-based P controller introduces a steady state error, because k_P_, in Equation (13), tends asymptotically to 1 when k_Static_ is increased. However, it can never reach 1. In the case of the higher spike temporal width of 10.1 μs, k_Static_ is very high, and consequently the steady state error (green in Figure 16(c)), has a low value. But in the case of low spike temporal width (8.2 μs), the steady-error is significantly increased (in purple), because k_P_ presents a value about 0.6.

As traditional continuous controllers introduce an integral (I) component to avoid the steady state error, now we are going to introduce the SI&G, implementing a spike-based PI controller. Using Equation (12) and assuming that STD(s) = 0, we can tune the spike-based PI controller, adjusting the SI&G gain, in (9), and setting the spike temporal width, in Equation (4). We must take into account, as Equation (12) indicates, that the spike temporal width affects not only the P component (k_P_), but also the equivalent integral component (k_I_). The result of applying a spike input step to the spike-based PI controller is presented in Figure 16(d). This plot contains the reconstruction of the spike frequency that flows inside the controller. Colored in blue, we can see the input spike frequency; in green, those spikes that represent the DC motor speed; in red, the DC motor speed error; in yellow, the SI&G output or integration error and, finally, in black, the combination of error and SI&G spikes, which will be expanded and applied to the DC motor. In this case, the DC motor speed presents a sub-damped response, presenting an overshoot; however, the steady state error has been removed. As it is expected from a continuous PI controller, the inclusion of the SI&G has added an integral component, which integrates the error until the steady state error is removed. However, integrators and also SI&G introduce a 90° phase lag that leads the DC motor to present sub-damped responses.

Finally, we have implemented the complete spike-based PID controller, in Figure 2. For this controller we have allowed every component to work (both SI&G and STD). This controller can be tuned using Equation (12), and in addition to the spike-based PI controller, the derivative gain, k_D_, can be set using Equations (4) and (11), as Equation (12) indicates. Figure 16(e) shows the controller spike frequency reconstruction for every signal between the controller components. The meanings of these signals are: in blue, spike input stream; in green, DC motor speed and, in red, controller error. Then, like in previous plots, we show in clear blue the SI&G output spikes, in purple, the STD output spikes, in yellow, the addition of SI&G and STD spikes, and finally, in black, the addition of error, SI&G and STD spikes, which will be expanded and applied to the DC motor. The DC motor response presents a sub-damped behavior; however, in this case, due to the addition of a derivative term, the DC motor overshoots. Settle time is lower when compared to the spike-based PI controller, but presenting a similar rise time.

In the last plot, Figure 16(f) shows the AER events that have been used to reconstruct the spike frequency. In this plot, the X axis represents time and the Y axis the AER events addresses. When an AER event is fired, a blue dot is added at its address with a temporal resolution of 0.2 μs. Figure 16(f) shows the different addresses that we have assigned to every signal inside the controller that can fire a spike. For example, addresses 0 and 1 represent the controller input spike or speed reference; addresses 2 and 3 represent the DC motor speed spikes, *etc*. Thanks to AER and timestamps, we are able to reconstruct the spike activity of the different elements of the controller and thus we are able to monitor and debug the controller behavior. We would also be able to propagate the controller state to other AER-based neuro-inspired layers that could react to this information. This will be done in future studies.

## 5. Spike-Based PID Controller Resources and Power Consumption

In order to determine the hardware requirements of the spike-based PID controller, we have synthesized different controllers for a low cost FPGA like the Spartan 3 included in the AER-Robot board. From a very general point of view, FPGA resources can be classified in two groups: general purpose logic programmable cells, which are known in Xilinx FPGAs as slices, and dedicated resources, as embedded multipliers, integrated memory, or oscillators. Previously presented spike-based processing blocks (which are integrated in our spike-based PID controller) are implemented using simple counters, without the need of multipliers to perform arithmetic operations, or a memory to store digital samples or a control algorithm, as usually needed by digital controllers. This feature is very interesting, because avoiding the need of FPGA dedicated resource usage allows us to replicate the spike-based processing system until we fill the general purpose slices of an FPGA. This is critical in low cost FPGAs as, for example, XC3S400 has only eight integer multipliers. Since the spike-based PID controller does not use dedicated resources, we focus in general purpose FPGA slices. We have synthesized spike-based PID controller for a different number of bits (N) in the SI&G and STD blocks, because these parameters have a direct influence in digital counters and comparators included in these blocks. It is clear that the higher the number of bits, the higher the usage of general purpose FPGA slices will be.

Table 2 shows brief information from the synthesis reports. The first two columns contain the number of bits of SI&G and STD used for synthesis, the next columns present the number of slices consumed, the percentage of FPGA usage and the maximum number of controllers that can be allocated inside an FPGA. The last column contains the maximum spike-based PID controller working frequency. The number of bits used for synthesis varies from 14 to 18 bits. The results in Table 2 clearly evidence that the number of slices clearly depends on the number of bits used in controller members, consuming from 120 to 148 slices, and operating from about 134 MHz, to 116 MHz, respectively. Slice requirements for a single controller are low, about 3.5% of our FPGA. This is possible thanks to the spike processing blocks simplicity. In part, low hardware consumption is due to the spike rate information codification, because we do not need to represent information with numerical values, which should be allocated in memory and demand high speed wide buses for data exchange. Another important aspect is the absence of an algorithm or running program. The presented spike-based PID controller is a completely reactive computational system; there is no thread running, program counter (PC), or stack. The system only reacts to spikes, saving the hardware that is needed to implement a processor for computational instruction flow management. Therefore, we are able to replicate, in average, 28 spike-based PID controllers in a single low cost FPGA, working fully in parallel, without the need of dedicated resources (multipliers). We also avoid multiplexing resources that can lead to bottlenecks (e.g., memory).

In [33] a PID controller for FPGA is presented. This controller is based on the space-vector-PWM (SVPWM) algorithm and requires 167 slices of a Spartan 3 200 FPGA. SVPWM synthesis report indicates a maximum clock frequency operation of 145 MHz. Nevertheless SVPWM requires a minimum number of 166 clock cycles for each PWM period, what implies 875 KHz maximum frequency and 6,885 ns of resolution for the duty-cycle.

Table 2 shows the power consumption of the Spike-based PID controller when running on a Spartan 3 400 alone. When synthesized several of them in the same FPGA, the power is not linearly incremented. In fact, there is almost no difference between one and two PID controllers. A commercial chip from Texas Instrument (UCD9222) integrates a PID controller with three phases and three poles. The power consumption of this commercial chip is around 130–150 mW for the controller, while our PID, implemented on a non-low power FPGA requires 65 mW for the 18 bit resolution PID.

Regarding to a multi-motor controller embedded on the same FPGA, we have synthesized a maximum number of 29 PID in a Spartan 3, 400 and 600 on a Spartan 6 LXT 150. This implies that when using a FPGA bigger enough, it is possible to include not only the motor controllers, but also other previous layers of processing, like gesture library, for example.

## 6. Conclusions

In this paper we have introduced a new approach that allows the implementation of analog like spike-based PID controllers on FPGAs. Spike-based PID controllers have been analytically analyzed and characterized, indicating that they are very close to continuous controller models, unless they are implemented in a completely digital device as FPGAs. For testing purposes we have implemented the spike-based controller in an AER developing platform, the AER-Robot, several experiments have been made in order to verify theoretical equations and different spike processing building blocks behaviors. These controllers are able to operate in a massively parallel fashion as they do not require dedicated hardware and a large number of them can be easily implemented in a low cost FPGA. The resource usage is much lower than using a conventional DSP approach and the system does not require the specific individual unit tunings needed in analog controllers.

## Figures and Tables

**Table 1.** Features of DC motor experiments.

Parameter	Value	Units
Nominal voltage	12	V
Nominal Speed	726.3	rads/s
Mechanical Time constant	176.1	ms
0dB Frequency	3.77	kHz
Terminal resistance	2.5	Ohm
Terminal inductance	0.2276e−3	H
Torque constant	13.9e−3	Nm/A
Speed constant	13.9e−3	V/(rads/s)
Rotor inertia	14e−7	kg·m^2^
Friction coefficient	1.48e−5	Nm/(rads/s)
Gear ratio	1:13	-
Quadrature encoder resolution	500	pulses per turn
Quadrature encoder max. frequency	500	kHz

**Table 2.** Hardware Resources Requirements and power consumption.

SI&G N bits	STD N bits	Number of slices	XC3S400 utilization (%)	Max. controllers number in XC3S400	Max Freq (MHz)	XPower (mW)
14	14	120	3.3482	29	134.35	60
14	16	127	3.5435	28	125.79	61
16	14	127	3.5435	28	125.82	61
16	16	133	3.7109	26	119.41	62
18	18	148	4.1295	24	116.75	65
